# Revisiting B-cell targeted therapies in rheumatoid arthritis: from paradoxical biology to deep immune reset

**DOI:** 10.3389/fimmu.2026.1809668

**Published:** 2026-05-04

**Authors:** Min Huang, Fangbing Dong, Qiaomei Liu, Shaofang Lin

**Affiliations:** Affiliated Calmette Hospital of Kunming Medical University/Kunming First People’s Hospital, Kunming, China

**Keywords:** anti-citrullinated protein antibody/ACPA, B-cell targeted therapies, deep immune reset, rheumatoid arthritis, rituximab

## Abstract

Targeted B-cell depletion via the anti-CD20 monoclonal antibody rituximab fundamentally altered the therapeutic algorithm for rheumatoid arthritis (RA). Despite its clinical entrenchment, approximately 40% of patients exhibit primary or secondary non-response, exposing critical limitations in conventional depletion strategies. This review critically deconstructs the mechanisms dictating therapeutic resistance and re-evaluates B-cell pathobiology through high-resolution transcriptomic and clinical cohort data. We challenge the monolithic perception of B-cell pathogenicity by detailing the emergence of protective, tolerogenic anti-citrullinated protein antibody (ACPA) clones (e.g., mC03, tACPA) and regulatory B cell (Breg) networks that actively suppress Th17 proliferation and Neutrophil Extracellular Trap (NET) formation. Mechanistic failure of rituximab is subsequently mapped to three biological evasions: the survival of CD20-negative plasmablasts within fortified synovial niches, the temporal-spatial persistence of highly mutated B-cell receptor (BCR) clonotypes, and the inadvertent eradication of IL-10/Granzyme B-producing Bregs, precipitating inflammatory rebound. Translating these molecular insights into clinical practice, we analyze the updated EULAR and ACR guidelines, defining the precise positioning of rituximab in high-risk patient strata, specifically those burdened with interstitial lung disease (RA-ILD) or recent malignancies. Finally, we evaluate the paradigm-shifting transition from superficial peripheral depletion to the “deep immune reset” orchestrated by CD19-directed Chimeric Antigen Receptor (CAR) T-cell therapy. Early clinical data validate that CAR-T cells actively penetrate solid tissues, collapse the follicular dendritic cell network, and eradicate long-lived autoreactive memory compartments, offering a tangible trajectory toward drug-free remission in multidrug-refractory RA.

## 1 Introduction

Rheumatoid arthritis (RA) operates as a chronic, systemically destructive autoimmune disorder characterized by symmetric polyarticular synovial inflammation, progressive cartilage degradation, and irreversible osteoarticular erosion (1–3). Historical conceptualizations of RA immunopathogenesis predominantly centered on T-cell-driven and macrophage-mediated cascades, relegating the B lymphocyte to a peripheral role as a mere precursor to autoantibody-secreting plasma cells (4). The clinical success of rituximab, a chimeric murine/human IgG1 monoclonal antibody targeting the CD20 surface antigen, irrevocably shattered this reductionist view. The rapid attenuation of synovial inflammation following rituximab-induced B-cell depletion established the B lymphocyte as a central, indispensable architect of RA pathology. These cells drive disease not only through autoantibody production but via potent antigen presentation and the localized secretion of pro-inflammatory cytokines, including interleukin-6 (IL-6) and tumor necrosis factor-alpha (TNF-α) (5, 6).

Prolonged clinical use of rituximab has revealed substantial therapeutic limitations. Observational cohorts and clinical studies indicate that up to one-third of patients fail to achieve a meaningful clinical response, and sustained remission remains unattained in a considerably larger proportion of treated individuals (7–9). Although many initial responders benefit substantially from rituximab, disease relapse commonly emerges following B-cell repopulation, necessitating repeated retreatment cycles in many patients to sustain low disease activity (10). This persistent therapeutic failure forces a critical re-evaluation of the binary hypothesis equating peripheral depletion with clinical remission.

High-resolution spatial transcriptomics, RNA-based next-generation sequencing, and advanced flow cytometry recently illuminated a profound duality within the RA immune landscape (3, 11). The humoral compartment orchestrates highly specific, counter-regulatory networks designed to restore homeostasis (12). Concurrently, the mechanisms of rituximab resistance are now recognized as complex immunobiological phenomena governed by tissue-specific cellular states and phenotypic evasion, rather than simple pharmacokinetic limitations (13, 14).

This review exhaustively synthesizes primary experimental and clinical evidence from the past decade to redefine the role of B-cell targeted therapies in RA. We dissect the paradoxical protective functions of specific autoantibodies, elucidate the molecular mechanisms of rituximab evasion within synovial sanctuaries, and rigorously map the strategic clinical positioning of anti-CD20 therapy against the latest international guidelines. Ultimately, we analyze the vanguard of cellular immunotherapy—CD19 CAR-T cells—evaluating how the transition from passive monoclonal antibodies to active, tissue-penetrating cellular agents facilitates a profound “deep immune reset,” offering unprecedented curative potential for refractory autoimmunity (15, 16).

## 2 Methods

To capture the contemporary paradigm shift in RA B-cell biology and therapeutic positioning, a systematic literature search was executed utilizing the PubMed, Embase, and Cochrane Library databases (3). The search strategy combined Medical Subject Headings (MeSH) and specific keywords, including (“Rheumatoid Arthritis” OR “Autoimmune Rheumatic Diseases”) AND (“Rituximab” OR “Anti-CD20” OR “B-cell depletion” OR “Regulatory B cells” OR “Protective ACPA” OR “CAR-T cell therapy”). To ensure the inclusion of the most advanced mechanistic insights and clinical data, the primary search was restricted to articles published between January 2020 and March 2026, with the inclusion of highly impactful foundational primary research dating back to 2018. Prioritization was strictly assigned to primary experimental studies (*in vivo* murine models and human ex vivo transcriptomic analyses), randomized controlled trials (RCTs), massive nationwide registry cohorts, and formal clinical practice guidelines (EULAR 2022/2023 and ACR 2021/2023 updates) (15, 17). Secondary reviews and non-peer-reviewed preprints were systematically excluded unless providing unique, highly specific mechanistic schematics directly relevant to the core thesis.

## 3 The paradoxical role of B cells in RA pathobiology

**Figure 1:**
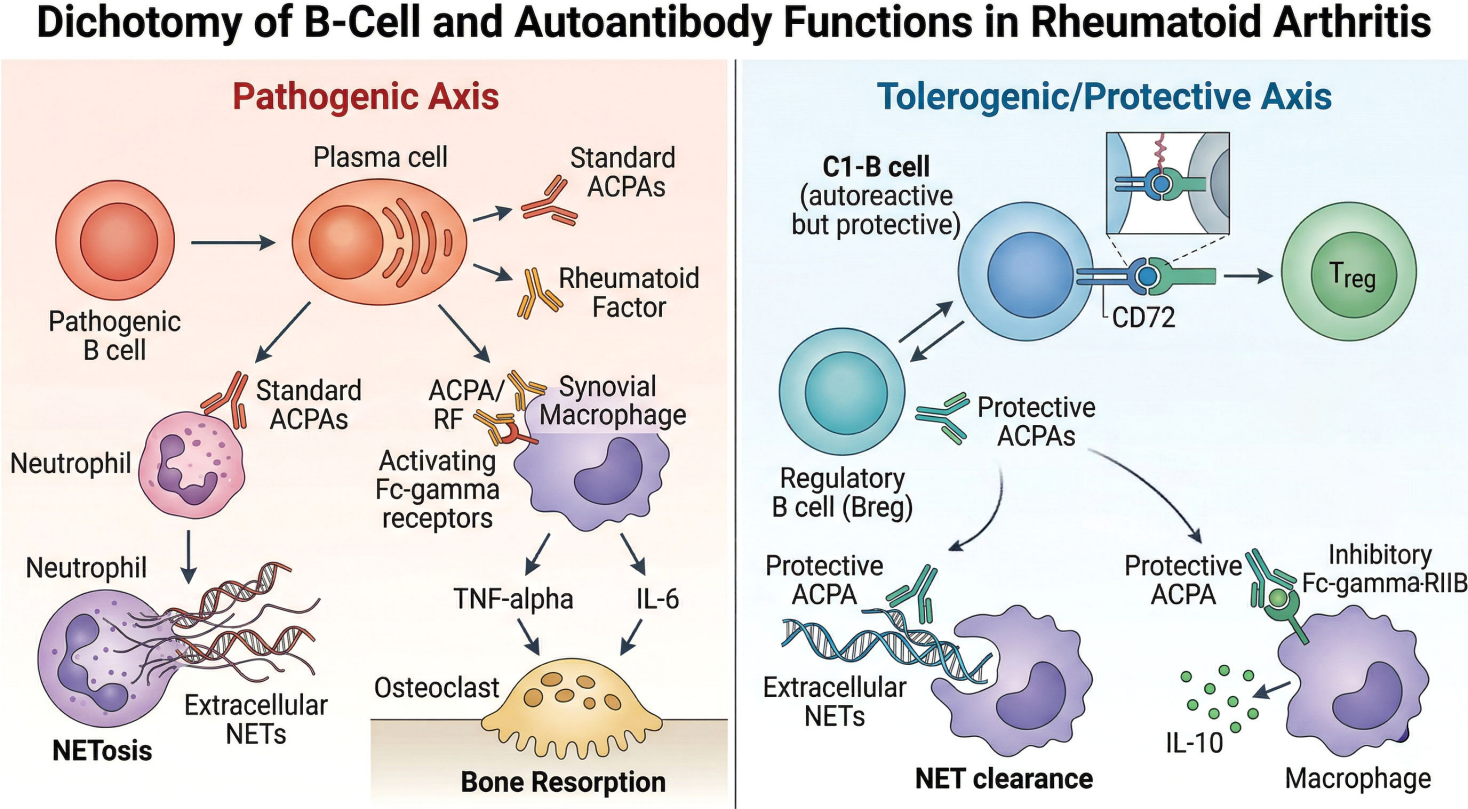
The immunological dichotomy of B cells and autoantibodies in rheumatoid arthritis. Historically viewed exclusively as drivers of pathogenesis, recent transcriptomic and functional data delineate a profound functional bifurcation within the humoral compartment. (Left Panel: Pathogenic Axis) Classical autoreactive B cells differentiate into plasma cells secreting pathogenic anti-citrullinated protein antibodies (ACPAs) and rheumatoid factor (RF). These autoantibodies form immune complexes that engage activating Fc-gamma receptors (FcγRs) on macrophages, triggering the release of pro-inflammatory cytokines (TNF-α, IL-6), promoting osteoclastogenesis, and stimulating neutrophils to undergo NETosis, thereby perpetuating the inflammatory cascade. (Right Panel: Tolerogenic/Protective Axis) A distinct network of protective responses actively suppresses joint destruction. Specific, highly mutated monoclonal ACPAs (e.g., mC03, E4 clones) physically opsonize pre-existing Neutrophil Extracellular Traps (NETs) for rapid macrophage clearance (efferocytosis) and preferentially bind the inhibitory receptor FcγRIIB on synovial macrophages, inducing massive IL-10 secretion and halting osteoclast differentiation. Concurrently, specialized Regulatory B cells (Bregs) and natural autoreactive C1-B cells actively present autoantigens to T cells via specific checkpoint axes (such as CD72 or PD-L1), suppressing Th17 proliferation and inducing the expansion of protective FoxP3+ Regulatory T cells (Tregs).

The immunological conceptualization of RA conventionally relies on a singular, pathogenic narrative. Anti-citrullinated protein antibodies (ACPAs) and rheumatoid factor (RF) operate as the primary molecular engines driving synovial inflammation and osteoclastogenesis (18, 19). Advanced recombinant antibody technologies and single-cell transcriptomics establish that the immune response in RA encompasses highly specific, counter-regulatory networks designed to restore homeostasis (20). As illustrated in **Figure 1**, this fundamental dichotomy contrasts classical pathogenic pathways with newly identified tolerogenic B-cell mechanisms, fundamentally altering the rationale for broad-spectrum B-cell depletion.

### 3.1 The protective efficacy of monoclonal ACPAs

The transition from asymptomatic autoantibody positivity to clinically overt inflammatory arthritis represents a complex immunological threshold (21). The total ACPA pool within a patient exhibits massive heterogeneity, comprising diverse clones with differing fine specificities, affinities, and Fc-domain glycosylation profiles (22). While a significant fraction drives immune complex deposition and complement activation, recent *in vivo* isolations demonstrate that specific monoclonal ACPA clones definitively ameliorate joint inflammation (20).

A systematic deconstruction of ACPA uniform pathogenicity evaluated eight distinct human-derived monoclonal ACPA clones in the collagen antibody-induced arthritis (CAIA) murine model (23). Rather than exacerbating pathology, the study revealed a clone-specific functional spectrum. Clones designated mC03 and mBVCA1 completely inhibited macroscopic arthritis development when administered prophylactically (24). When the mC03 clone was administered therapeutically at the peak of clinical disease (day 8), treated subjects exhibited near-complete recovery from joint inflammation within 48 hours. Competitive *in vivo* experiments confirmed that the protective effects of mC03 operate dominantly over pathogenic clones. Mechanistic dissection proved that this anti-inflammatory capacity relies strictly on the Fc-gamma receptor (FcγR) network. Cleaving the antibody to utilize only F(ab’)2 fragments, or engineering FcγR-null mutations, abolished the suppressive effect, indicating direct inhibitory engagement with cellular Fc receptors overriding immune complex-generated activating signals (24, 25).

Corroborating these findings, single-cell sequencing of the active RA plasmablast repertoire identified multi-specific recombinant ACPAs exhibiting robust binding affinities for native peptidylarginine deiminase 4 (PAD4) and citrullinated histones (26). Early-phase administration of these clones in CAIA models significantly reduced disease severity across the treated cohort, suggesting a preventive interception of the inflammatory cascade prior to irreversible synovial fibroblast hyperproliferation and massive neoangiogenesis (23).

Further cellular resolution identifies the active abrogation of Neutrophil Extracellular Traps (NETs) as a primary protective mechanism. NETs supply a continuous source of externalized citrullinated autoantigens, perpetuating the breach of immune tolerance (27). Recent structural analyses isolated a highly specific monoclonal ACPA (tACPA) that physically disrupts this cycle (28). Unlike pathogenic ACPAs that stimulate NETosis, tACPA actively inhibits NET formation and binds pre-existing extracellular NETs. This specific opsonization process fundamentally alters the biochemical architecture of the NETs, facilitating their rapid recognition, engulfment, and clearance (efferocytosis) by synovial macrophages (27). Systemic tACPA administration in acute CAIA mouse models reduced ultimate arthritis clinical scores by 94% compared to untreated disease controls (29).

Similarly, the E4 clone (an hIgG1 isotype) directs anti-inflammatory macrophage polarization. The E4 antibody forms specialized immune complexes specifically with citrullinated alpha-enolase, a protein highly abundant in inflammatory synovial fluid (30). Rather than binding activating Fc receptors (such as FcγRIIIa), E4-immune complexes preferentially engage the inhibitory receptor FcγRIIB on synovial macrophages. This cross-linking phosphorylates the intracellular Immunoreceptor Tyrosine-based Inhibitory Motif (ITIM), recruiting phosphatases that rapidly terminate pro-inflammatory signaling cascades (31). This engagement prompts massive localized secretion of interleukin-10 (IL-10) and potently suppresses the differentiation of synovial macrophages into bone-resorbing osteoclasts, directly preserving subchondral bone integrity and halting the erosive phase of the disease (20, 32).

### 3.2 Autoreactive B cells and Bregs as T-cell suppressors

The second pillar of the RA paradigm shift involves the regulatory capacity of the B lymphocyte compartment. Clinical anomalies, such as severe disease flares occasionally observed during rituximab therapy or upon immune reconstitution, are now mechanistically linked to the unintended eradication of Regulatory B cells (Bregs) and specific, tolerance-inducing autoreactive B cells (33, 34).

Groundbreaking research overturned decades of assumptions regarding autoreactivity by identifying a ubiquitous population of natural autoreactive B cells specific for a defined triple-helical epitope (C1) on collagen type-II (35). These C1-B cells reside within physiological repertoires but are profoundly diminished (an eight-fold decrease) in patients with active RA (35). Adoptive transfer of a highly limited number of magnetically enriched C1-specific B cells into autoimmune-prone recipient mice completely stunted the development of autoimmune arthritis. Transcriptomic single-cell sequencing revealed that upon migrating to joint-draining lymph nodes, C1-B cells capture self-antigens and present them directly to infiltrating T cells. Instead of activating pathogenic Th1 or Th17 responses, they induce the massive expansion and activation of COL2-reactive Regulatory T cells (Tregs) (36). This potent Treg induction requires contact-dependent engagement via the inhibitory C-type lectin CD72; specific *in vivo* blockade of CD72 completely impeded Treg expansion and fully reversed the arthritis suppression, proving CD72 operates as a critical downstream suppressor mechanism regulating antigen-specific T cell tolerization (35, 37).

Parallel Breg populations leverage established immune checkpoint pathways. PD-L1+ B cells, significantly reduced in the peripheral circulation of untreated RA patients compared to healthy controls, robustly upregulate surface PD-L1 upon TLR9 engagement and IL-2 stimulation (38). Co-culture assays prove these PD-L1+ B cells potently suppress CD8+ T cell proliferation and abrogate the intracellular production of highly pro-inflammatory, tissue-damaging cytokines, specifically Tumor Necrosis Factor (TNF) and Interferon-gamma (IFN-γ) (38). The introduction of an anti-PD-L1 blocking monoclonal antibody significantly reversed the suppression of both CD8+ T cell proliferation and cytokine production, confirming an evolutionarily tailored mechanism modulating cytotoxic T cell responses increasingly recognized for driving synovial inflammation (39).

Environmental factors, notably the diverse composition of the gut microbiome, directly modulate Breg functionality. Dietary supplementation with specific short-chain fatty acids (SCFAs), most notably butyrate, fundamentally alters the host’s gut microbiota, significantly increasing the systemic circulatory production of 5-Hydroxyindole-3-acetic acid (5-HIAA) (40). 5-HIAA operates as a potent, highly specific ligand for the Aryl-Hydrocarbon Receptor (AhR) highly expressed internally by regulatory B cells. AhR activation triggers profound transcriptional and metabolic reprogramming within these Bregs, aggressively upregulating the production and secretion of IL-10 (41). This amplified IL-10 axis efficiently attenuates CD4+ T cell proliferation throughout the lymphatic system and robustly inhibits the downstream production of pro-inflammatory cytokines by effector T cells that would otherwise migrate to the joints (42).

Furthermore, B cells actively modulate the local inflammatory microenvironment through highly specific enzymatic activity. The ectonucleotidase CD39, expressed on a specialized subset of B cells, operates consecutively with CD73 to hydrolyze highly pro-inflammatory extracellular ATP (eATP) into adenosine (ADO) (43). Adenosine acts as a molecule with profound immunosuppressive properties, rapidly binding to A2A receptors on T cells to increase intracellular cAMP and halt proliferation. Activation of CD19+ B cells from therapy-responding RA patients generates sufficient adenosine to significantly decrease the proliferation of both CD4+ and CD8+ T cells, drastically reducing the frequency of TNF-producing effector T cells (44, 45). Longitudinal clinical analyses validate that sustained post-treatment CD39 upregulation on circulating B cells correlates directly with lowered Disease Activity Scores (DAS28) and reduced systemic levels of pathogenic ACPAs (45).

Finally, a highly distinct mechanism of T-cell suppression involves the secretion of cytotoxic granules by B cells. While Granzyme B (GrB) is traditionally considered the exclusive functional domain of Natural Killer (NK) cells and Cytotoxic T Lymphocytes (CTLs), recent primary research establishes the existence of GrB-producing regulatory B cells (46). These GrB-producing Bregs actively suppress the proliferation of pathogenic Th1 and Th17 cells by proteolytically cleaving the T cell receptor (TCR) zeta chain, rendering the T cell functionally inert to antigen presentation (47). In cases of severe hyperactivation, these Bregs directly induce apoptosis in CD4+CD25− effector T cells, physically eliminating the pathogenic threat from the microenvironment. The pathologically diminished frequency of these protective GrB-producing Bregs in active RA correlates directly with elevated systemic autoantibody titers and increased tender and swollen joint counts (46). The diverse characteristics, specific markers, and suppressive mechanisms of these newly identified protective autoantibodies and regulatory B cell subsets are comprehensively summarized in **Table 1**.

**Table 1:** Characteristics of protective autoantibodies and regulatory B cell (Breg) subsets in rheumatoid arthritis.

Cellular/molecular target	Identifying markers/specificity	Primary suppressive mechanism	Effector output	Clinical relevance in RA	Ref.
Monoclonal ACPA (mC03 clone)	Broad citrullinated reactivity	FcγR-dependent dominant suppression (complement independent)	Overrides pathogenic immune complex signaling	Completely inhibits macroscopic arthritis prophylactically and therapeutically	(23, 25)
Therapeutic ACPA (tACPA)	Citrullinated NET components	Direct binding and physical disruption of pre-existing NETs	Facilitates rapid macrophage efferocytosis	Abrogates NETosis cycle; reduces clinical arthritis score by 94%	(29)
Autoreactive C1-B Cells	CD72+, COL2-specific BCR	Captures autoantigens for tolerogenic presentation to T-cells	Induces massive expansion of FoxP3+ Tregs	Profoundly diminished in active RA; adoptive transfer stunts arthritis	(36, 38)
AhR-Activated Bregs	Aryl-Hydrocarbon Receptor (AhR)+	Activated by gut microbiota-derived metabolites (5-HIAA)	Aggressive upregulation of IL-10 secretion	Attenuates CD4+ T-cell proliferation; restricts pathogenic plasmablast differentiation	(43, 44)
Purinergic Bregs	CD19+CD39+CD73+	Sequential hydrolysis of pro-inflammatory eATP	Generates highly immunosuppressive adenosine (ADO)	Decreases TNF-producing effector T-cells; tracks with therapy response	(46, 48)
Granzyme B-Producing Bregs	CD19+Granzyme B (GrB)+	Proteolytic cleavage of TCR zeta chain; direct apoptosis induction	Physically eliminates hyperactivated CD4+CD25- T-cells	Loss of subset correlates with elevated autoantibody titers and erosive damage	(49, 50)

This table delineates the paradigm shift from global B-cell pathogenicity to targeted immunoregulation. Protective clones and specific Breg subsets operate via distinct contact-dependent and soluble pathways to enforce peripheral tolerance. ACPA, Anti-Citrullinated Protein Antibody; AhR, Aryl-Hydrocarbon Receptor; BCR, B Cell Receptor; Breg, Regulatory B Cell; eATP, Extracellular Adenosine Triphosphate; FcγR, Fc-gamma Receptor; NET, Neutrophil Extracellular Trap; TCR, T Cell Receptor; TNF, Tumor Necrosis Factor; Treg, Regulatory T Cell.

## 4 Decoding the mechanisms of anti-CD20 failure

Intravenous rituximab administration reliably achieves profound clearance of peripheral blood B cells, frequently reducing the circulating CD20+ compartment by greater than 95% within weeks (48). Despite this near-total peripheral clearance, the clinical efficacy of the agent remains highly variable. High-sensitive flow cytometry (HSFC) and advanced spatial transcriptomics confirm that therapeutic failure is a complex immunobiological phenomenon governed by tissue-specific cellular states, evolutionary escape mechanisms, and phenotypic evasion, rendering simplistic pharmacokinetic explanations completely obsolete (13, 49).

### 4.1 Clonal dominance and spatial sanctuaries

The B Cell Receptor (BCR) repertoire provides a highly specific molecular fingerprint of the adaptive immune compartment. Formed via V(D)J recombination and somatic hypermutation (SHM) within germinal centers, the BCR repertoire reflects the unique clonal expansions responding to autoantigens in RA (50). RNA-based next-generation sequencing evaluating the spatial and temporal dynamics of the BCR repertoire unequivocally ties clinical non-response to an incomplete disruption of the dominant, pre-existing BCR repertoire (7, 50).

In patients achieving a moderate or good EULAR clinical response, rituximab successfully eradicates dominant B cell clones from the peripheral blood. Conversely, at merely four weeks post-infusion, non-responders harbor a significantly higher number of dominant clones (median 36 vs. 18 in responders, p<0.01) (51). When assessing “clonal overlap”—the percentage of identical BCR sequences found in both baseline and week 4 samples—non-responders demonstrate a median overlap of 5%, whereas responders show a total 0% overlap (52). The surviving repertoire exhibits a significantly increased somatic mutation load within IGHV genes, shifting from a median of 0.012 mutations/bp at baseline to 0.056 mutations/bp post-treatment (53). This elevated mutation load identifies mature memory B cells and plasmablasts, proving that rituximab selectively fails to clear the most pathogenically advanced, highly adapted compartments of the humoral immune system (54).

Crucially, a severe spatial and temporal disconnect characterizes rituximab’s efficacy. While profound shifts in clonal dominance and mutation load occur rapidly in the peripheral blood, the BCR repertoire within corresponding synovial tissue biopsies remains virtually unchanged at week 4. Significant reductions in synovial clonal overlap are not detectable until week 16 post-treatment (7, 55). This massive temporal lag indicates that synovial B cells are structurally and biochemically insulated from the immediate systemic cytotoxicity of intravenously administered rituximab.

This synovial sanctuary is actively orchestrated by ectopic lymphoid structures (ELS) (56). Specialized cell types, including synovial fibroblast-like synoviocytes (FLS) and unique LYVE1+CD206+ perivascular tissue-resident macrophages, saturate the niche with powerful B-cell survival cytokines: BAFF (B-cell activating factor) and APRIL (56). This receptor ligation triggers non-canonical NF-κB intracellular signaling, forcibly upregulating potent anti-apoptotic proteins, such as Bcl-2 (57). Exacerbating this resistance, the systemic lysis of peripheral B cells by rituximab removes the primary consumer of circulating BAFF. This induces a massive surge in free BAFF levels, acting as a survival hyper-stimulant for remaining tissue-resident memory B cells, granting them supra-physiologic resistance against lingering apoptotic signals (58). Concurrently, surviving tissue-resident B cells upregulate complement-inhibitory membrane proteins (CD55 and CD59), neutralizing complement-dependent cytotoxicity (CDC) and rendering the antibody functionally inert against the synovial compartment (14, 59).

### 4.2 Phenotypic evasion: CD20-negative plasmablasts and plasma cells

The target antigen, CD20, is not uniformly expressed throughout the lifecycle of a B lymphocyte. While highly expressed on naive and memory B cells, CD20 is systematically downregulated and lost entirely as an activated B cell terminally differentiates into a plasmablast and long-lived plasma cell (60). Rituximab’s absolute dependence upon physical CD20 engagement renders these mature antibody factories entirely invisible to and invincible against the therapy (61).

Advanced multi-parameter flow cytometry consistently verifies that RA patients harboring higher frequencies of circulating CD20-negative plasmablasts prior to therapy, or exhibiting immediate spikes in these cells post-treatment, demonstrate dramatically elevated odds ratios for clinical relapse and failure to achieve LDA (62, 63). Histopathological analyses of arthroscopic synovial biopsies obtained before and after rituximab therapy demonstrate that while subsynovial CD20+ infiltrating B cells diminish, the local population of CD138+ plasma cells frequently remains robust and unaltered (13, 59).

Furthermore, the bone marrow serves as the apex sanctuary for long-lived, CD20-negative plasma cells. Unique microenvironmental niches heavily fortified with CXCL12 (SDF-1) sustain the lifespan of plasma cells indefinitely, entirely independent of ongoing B-cell receptor stimulation (64, 65). Experimental profiling demonstrates that marrow plasma cell populations persist completely unfazed, even when upstream CD20+ precursor pools are eradicated (64, 66). The clinical consequence is the uninterrupted, continuous synthesis of pathogenic IgG-rheumatoid factor (RF) and ACPA. These autoantibodies rapidly form massive immune complexes within the diarthrodial joint space, binding avidly to FcγRs on local macrophages and driving the unceasing secretion of matrix metalloproteinases (MMPs) and severe pro-inflammatory cytokines autonomously, requiring no input from the destroyed CD20+ B cells (67).

### 4.3 The immunoregulatory paradox: accidental depletion of Bregs

Indiscriminate B-cell depletion is fundamentally a double-edged sword. Rituximab functions as an entirely indiscriminate cytotoxic agent, possessing no capacity to distinguish between a pathogenic, autoreactive memory B cell and a protective, IL-10-secreting regulatory B cell (68, 69). Because the CD20 antigen is abundantly expressed on transitional B cells and the broader Breg compartment, rituximab infusion eradicates the protective regulatory network just as efficiently and violently as it eliminates the pathogenic drivers (70).

The accidental, total eradication of the Breg compartment fundamentally destabilizes T-cell homeostasis. Experimental murine models and clinical flow cytometry tracking indicate that the sudden removal of B-cell-derived IL-10 removes regulatory constraints, allowing for the unchecked proliferation and hyperactivation of autoreactive effector T cells, particularly the highly destructive Th17 subset (12, 71, 72). In subsets of RA patients whose underlying immunopathology is driven more by a failure of regulation rather than massive autoantibody overproduction, rituximab administration can paradoxically worsen specific inflammatory parameters, precipitating a severe clinical phenomenon known as “inflammatory rebound” (73). Moreover, by repeatedly clearing CD20+ precursor pools, chronic rituximab therapy starves the APRIL signaling pathway of the raw cellular materials necessary to generate new IgA+ IL-10-producing Bregs, locking the patient’s immune system into a state of chronic, irretrievable regulatory deficit (74).

### 4.4 Target-absence and synovial endotypes: the R4RA trial

The implicit assumption that B cells uniformly drive local inflammatory processes in all seropositive or anti-TNF-refractory patients was definitively dismantled by the identification of diverse synovial endotypes. Ultrasound-guided synovial biopsies categorize RA patients into three distinct pathotypes: lympho-myeloid (B-cell rich), diffuse-myeloid (macrophage-dominated), and fibroid (pauci-immune) (75).

The target-absence hypothesis was rigorously tested in the landmark R4RA (Response/Resistance to Rituximab versus Tocilizumab in Rheumatoid Arthritis) trial (76). The trial stratified patients based on an advanced RNA-sequencing-derived “B-cell molecular signature.” In the molecularly defined “B-cell poor” pathotype, tocilizumab (IL-6 receptor antagonist) demonstrated clear, statistically significant clinical superiority. The tocilizumab cohort achieved a 63% response rate for a 50% improvement in the Clinical Disease Activity Index (CDAI50%), whereas the rituximab cohort languished at a 36% response rate (p=0.035) (76).

The mechanistic implications are undeniable: treating a diffuse-myeloid or fibroid RA endotype with rituximab is biologically discordant. In these patients, the inflammatory engine is entirely driven by macrophages, dendritic cells, and hyper-proliferative fibroblasts, operating independently of the CD20+ B cell axis (13, 75). Rituximab non-response in this demographic is not a failure of the drug to deplete its target, but rather the administration of a highly specific targeted therapy into a microenvironment entirely devoid of its required antigen (76).

## 5 Clinical positioning and precision optimization in refractory RA

The realization that rituximab non-response is deeply rooted in synovial endotypes and microenvironmental sanctuaries necessitates a radical departure from rigid, step-up therapeutic sequencing. International clinical guidelines progressively reflect this mechanistic nuance. The European Alliance of Associations for Rheumatology (EULAR) 2022/2023 updates and the American College of Rheumatology (ACR) 2021/2023 guidelines transitioned the management of Rheumatoid Arthritis from generic palliation toward a heavily risk-stratified, precision-guided ‘Treat-to-Target’ (T2T) architecture (15, 77). Within these highly protocolized environments, rituximab occupies a distinct position, elevated not by generic efficacy but by its exceptionally targeted immunological profile in the presence of severe, life-limiting comorbidities.

### 5.1 EULAR and ACR algorithmic stratification

The T2T strategy mandates the immediate initiation of conventional synthetic disease-modifying antirheumatic drugs (csDMARDs), universally prioritizing methotrexate, to achieve sustained clinical remission or low disease activity (LDA) within six months (17). Upon csDMARD failure, the algorithmic pathways bifurcate based on regional regulatory constraints and patient risk profiles.

The ACR 2021 guidelines impose a highly specific restriction on the basal use of rituximab for the general RA population. Adhering strictly to United States Food and Drug Administration (FDA) labeling, the ACR relegates anti-CD20 therapy to the post-Tumor Necrosis Factor inhibitor (TNFi) salvage setting (17). Conversely, the EULAR 2022/2023 update categorizes rituximab as a fully equal-tier biological DMARD (bDMARD). EULAR explicitly endorses rituximab deployment immediately following Phase I csDMARD failure if the patient exhibits poor prognostic factors, including high autoantibody titers or early joint erosions (78).

This regulatory divergence collapses when evaluating high-risk patient strata. The publication of the ORAL Surveillance clinical trial definitively demonstrated higher incidence rates of major adverse cardiovascular events (MACE) and incident malignancies associated with Janus kinase (JAK) inhibitors compared to TNFi (79). Consequently, the EULAR task force explicitly altered the therapeutic hierarchy. Biological DMARDs, including rituximab, are now strictly prioritized over targeted synthetic DMARDs (JAK inhibitors) in any patient possessing baseline cardiovascular or malignancy risk factors (78). This forceful directive structurally elevates rituximab, bypassing entire classes of newer therapeutics based purely on physiological safety profiles.

### 5.2 Strategic deployment in RA-associated interstitial lung disease

Interstitial lung disease represents the most prognostically severe extra-articular manifestation of RA, conferring a three-fold increase in mortality risk (80). The clinical management of RA-ILD sits at a contradictory intersection: treating the severe articular components with standard DMARDs risks directly exacerbating the pulmonary fibrosis. Methotrexate carries a documented risk of hypersensitivity pneumonitis, while TNFi agents are repeatedly implicated in the paradoxical acceleration of interstitial fibrosis (81, 82).

To resolve this therapeutic ambiguity, the American College of Rheumatology and the American College of Chest Physicians (CHEST) released landmark, collaborative guidelines in 2023 specifically for the treatment of ILD in systemic autoimmune rheumatic diseases (83). These guidelines aggressively modify the standard T2T algorithm. For patients presenting with RA-ILD, the panel conditionally recommends rituximab as a first-line ILD treatment option, elevating the anti-CD20 monoclonal antibody to stand equally alongside traditional potent immunosuppressants like mycophenolate mofetil and intravenous cyclophosphamide (84). This strategic elevation deliberately circumvents the pulmonary toxicities of MTX and TNFi, recognizing rituximab’s dual capacity to simultaneously suppress devastating joint inflammation while independently stabilizing pulmonary decline (85).

Robust epidemiological data validate this aggressive guideline positioning. A 2022 systematic review and meta-analysis aggregating data across 15 distinct clinical cohorts (314 highly characterized patients) evaluated rituximab efficacy in RA-ILD (86). The pathophysiological hypothesis posits that B-cells drive lung fibrosis via the continuous production of autoantibodies that directly stimulate collagen-producing fibroblasts in the lung parenchyma. By chemically depleting CD20+ B-cells, rituximab dismantles this localized immune architecture. The meta-analysis yielded a pooled proportion of 88% (95% CI: 0.76 to 0.96) for patients demonstrating halted progression or clinical improvement in overall disease (86, 87). Quantitative metrics confirmed definitive physiological gains, including a 7.50% mean improvement in percent-predicted Forced Vital Capacity (FVC) and a 6.39% improvement in Diffusion Capacity for Carbon Monoxide (DLCO) following therapy (86).

### 5.3 Preserving immunosurveillance: the oncological exception

The intersection of severe RA and systemic oncology presents the most high-stakes decision-making matrix in modern internal medicine. Chronic systemic inflammation inherently elevates the baseline risk for developing malignancies in RA patients (88). When a patient develops a *de novo* malignancy or presents with a recent history of cancer, the profound immunosuppression required to control joint destruction directly conflicts with the biological necessity for robust immunosurveillance to prevent tumor recurrence.

Tumor Necrosis Factor is absolutely integral to the induction of apoptosis in mutated malignant cells and the activation of CD8+ cytotoxic T-cells directed against forming solid tumors (89). Pharmacological blockade of TNF is historically viewed by oncologists with intense suspicion, fearing a catastrophic unmasking of dormant micrometastases (90). Rituximab selectively targets the CD20 antigen. The biological lifting of solid tumor immunosurveillance is predominantly executed by the T-cell compartment and Natural Killer (NK) cells. Depleting the B-cell compartment does not systematically impair the body’s innate or adaptive cellular ability to detect and destroy aberrant epithelial cells.

This mechanistically sound theoretical advantage is rigorously validated by recent massive clinical datasets. A 2025 nationwide registry-based cohort study conducted in Denmark exhaustively investigated cancer recurrence risk in RA patients with a prior documented solid cancer in remission (90). Tracking 720 highly characterized patients utilizing inverse probability of treatment weighting, the researchers found absolutely zero evidence of increased cancer recurrence for RA patients treated with rituximab compared to those managed conservatively on csDMARDs. The calculated Hazard Ratio (HR) for rituximab was 0.94 (95% CI: 0.32 to 2.11), an entirely non-significant variance from the baseline risk (90).

Recognizing this unique dual efficacy—aggressively treating autoimmune joint destruction while systemically suppressing the cellular lineage responsible for specific malignancies—the ACR 2021 guidelines carve out an explicit, unconditional exception. For patients presenting with a history of lymphoproliferative disorders, rituximab entirely bypasses the mandatory TNFi failure requirement and is instantly elevated to a conditionally recommended primary biological DMARD (17). A detailed comparative analysis of rituximab’s algorithmic positioning across different clinical scenarios and risk profiles is provided in **Table 2**.

**Table 2:** Strategic algorithmic positioning of rituximab in RA: EULAR (2022/2023) vs. ACR (2021/2023) guidelines.

Clinical scenario	EULAR 2022/2023 positioning	ACR 2021/2023 positioning	Primary mechanistic/clinical rationale	Ref.
Phase I (csDMARD Naïve / Early Disease)	Not indicated. (MTX + short-term GC is mandated).	Not indicated. (MTX monotherapy strongly recommended).	Requires establishment of refractory state before broad systemic depletion.	(16, 17)
Phase II (Post-MTX Failure)	Available as an equal-tier first-line bDMARD if poor prognostic factors are present.	Excluded from consideration (unless specifically failed prior TNFi).	ACR strictly adheres to initial FDA labeling constraints; EULAR allows mechanistic flexibility.	(16, 86, 88)
Phase III (Post-TNFi Failure)	Highly Recommended. Cycling to a different MoA (anti-CD20) is strongly advised.	Highly Recommended. Standard entry point.	TNFi failure indicates a likely B-cell/autoantibody-driven endotype necessitating a class switch.	(85, 88)
Comorbidity: RA-ILD (Interstitial Lung Disease)	Highly Prioritized. Broadly favored over TNFi class.	Conditionally Recommended as First-Line (per 2023 ACR/CHEST joint guideline).	Avoids MTX pneumonitis and TNFi-induced fibrotic exacerbation; stabilizes pulmonary decline.	(94, 95)
Comorbidity: Recent Malignancy / High MACE Risk	Strictly Preferred over JAK inhibitors (due to ORAL Surveillance data).	Explicit Exception Granted. Bypasses TNFi requirement for lymphoproliferative history.	Transient CD20 depletion spares T-cell/NK-cell solid tumor immunosurveillance, demonstrating zero increased hazard ratio for recurrence.	(90, 91, 102)

Rituximab positioning diverges significantly based on international regulatory philosophy but forcefully converges when addressing severe, life-limiting comorbidities, highlighting its unique safety profile in precision oncology and pulmonology settings. bDMARD, Biological Disease-Modifying Antirheumatic Drug; csDMARD, Conventional Synthetic DMARD; FDA, Food and Drug Administration; GC, Glucocorticoids; JAK, Janus Kinase; MACE, Major Adverse Cardiovascular Events; MoA, Mechanism of Action; MTX, Methotrexate; RA-ILD, Rheumatoid Arthritis-Associated Interstitial Lung Disease; TNFi, Tumor Necrosis Factor Inhibitor.

## 6 Beyond depletion: the era of deep immune reset

**Figure 2:**
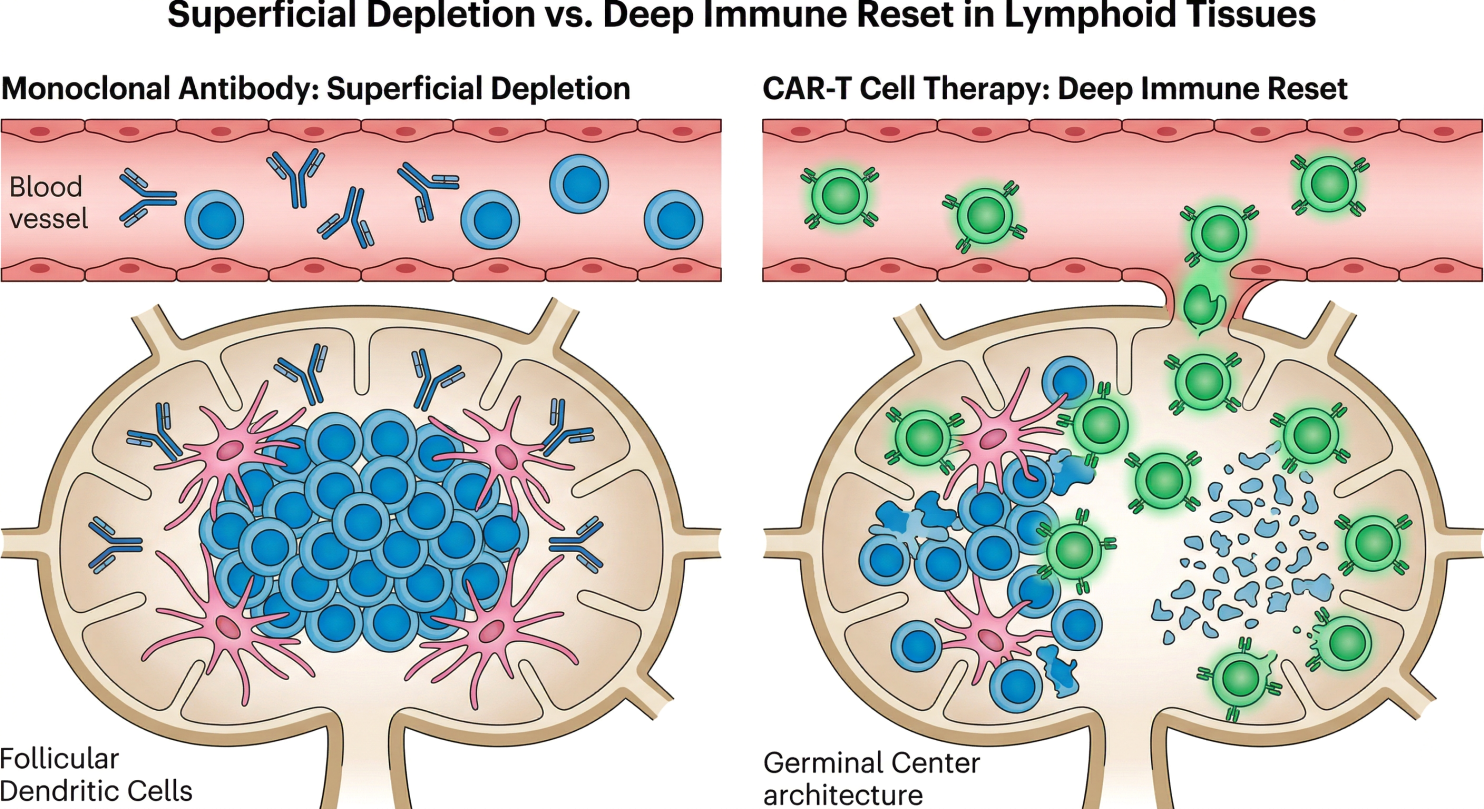
Superficial depletion versus deep immune reset in secondary lymphoid organs. The mechanistic dichotomy between passive monoclonal antibody therapy and active cellular immunotherapy defines long-term clinical outcomes in refractory autoimmunity. (Left Panel: Monoclonal Antibody/Rituximab) Large, passive IgG molecules effectively clear circulating CD20+ B cells within the intravascular compartment via complement and host-effector cell recruitment (CDC/ADCC). However, they fail to penetrate the dense architecture of secondary lymphoid organs and synovial niches. Tissue-resident memory B cells survive within the germinal center, protected by survival cytokines and a structurally intact Follicular Dendritic Cell (FDC) network, serving as a continuous reservoir for disease relapse. (Right Panel: CAR-T Cell Therapy) Autologous T cells engineered with Chimeric Antigen Receptors (CAR-T) operate as agile, actively migrating "living drugs." They extravasate from the peripheral circulation, physically penetrating deep solid tissues and lymphoid sanctuaries. CAR-T cells execute direct, perforin/granzyme-mediated cytotoxicity against all CD19-expressing cells. Crucially, because FDCs acquire CD19 antigens from B cells via trogocytosis, CAR-T cells inadvertently eradicate the supportive FDC network. This destruction causes the total architectural collapse of the pathogenic germinal center, permanently erasing the autoimmune immunological memory and facilitating a "deep immune reset.".

The widespread clinical failure of rituximab in a substantial cohort of seropositive, B-cell-rich RA patients forcefully terminates the era of passive, superficial B-cell depletion. The anatomical persistence of highly mutated memory B cells within fortified synovial niches, coupled with the phenotypic evasion of autoantibody-secreting plasmablasts, dictates a requirement for active, tissue-penetrating cellular interventions. The field is rapidly transitioning toward engineered living cellular therapies capable of executing a permanent, structural obliteration of the autoimmune memory compartment—a phenomenon defined as the “deep immune reset” (13, 91). **Figure 2** delineates the profound mechanistic dichotomy between passive monoclonal antibody depletion and active cellular eradication.

### 6.1 The architecture of CAR-T cell therapy in autoimmunity

Chimeric antigen receptor (CAR) T-cell therapy precipitates a paradigm shift in the treatment of multidrug-refractory autoimmune diseases. Originally developed for relapsed hematological malignancies, the therapy involves the ex vivo genetic modification of autologous T cells to express a synthetic receptor targeting a specific cell-surface antigen (16, 92). By redirecting T cells against the CD19 antigen—a ubiquitous transmembrane protein expressed significantly more broadly across the B-cell lineage than CD20, capturing early B cells and retaining expression longer on differentiating plasmablasts—CAR-T cells execute a direct, robust, and tissue-penetrating cytotoxic response (93).

Following fludarabine and cyclophosphamide preconditioning (acting as a “cytokine sink” to enhance engraftment), the infused CAR-T cells undergo massive logarithmic expansion *in vivo* (94). These agile, “living drugs” operate independently of host effector mechanisms. They actively extravasate across endothelial barriers, infiltrate deep solid tissues, and physically seek out target antigen-expressing cells, releasing cytolytic perforin and granzymes to bypass the protective shielding provided by local BAFF, APRIL, and complement-inhibitory proteins (95).

### 6.2 Clinical efficacy and the collapse of the germinal center

Between 2022 and 2026, an expanding compendium of clinical trial data, spearheaded by the Erlangen group and corroborated by massive multicenter basket trials (e.g., CASTLE and Breakfree-1), demonstrated that CD19-directed CAR-T cell therapy safely induces deep, sustained, and drug-free clinical remissions in patients with severe, treatment-refractory systemic lupus erythematosus (SLE), idiopathic inflammatory myopathies (IIM), systemic sclerosis (SSc), and RA (16, 92).

A seminal 2024 study published in the *New England Journal of Medicine* evaluated 15 patients treated with CD19 CAR-T cells across three distinct autoimmune diseases (92). With a median follow-up extended to 15 months, the study unequivocally demonstrated the sustainability of the therapeutic effect. All patients achieved rigorous clinical remission criteria, eradicating circulating autoantibodies and completely withdrawing from all baseline immunosuppressive drugs (92). Crucially, unlike the imperfect and variably relapse-associated B-cell repopulation observed after rituximab, reconstitution of peripheral B cells after CD19 CAR-T therapy did not necessarily trigger recurrent autoimmune disease in the reported cohort (67).

This clinical dichotomy is elegantly explained by the “deep immune reset.” Performing sequential, ultrasound-guided biopsies of secondary lymphoid organs, researchers directly compared the depth of B-cell depletion achieved by CD19-CAR T-cell therapy against that of rituximab (95). While rituximab achieved only superficial peripheral clearance, leaving median counts of 196 CD19+ cells/mm² in the lymph nodes, post-CAR-T biopsies confirmed absolute 0 B cells per mm² within the lymphatic architecture (95).

The secondary consequence of this deep tissue depletion is the total structural collapse of the B-cell maturation compartment. Germinal centers are maintained by an intricate signaling web between B cells, Follicular Dendritic Cells (FDCs), and T follicular helper (TFH) cells (96). Rituximab preserves these supportive networks entirely, leaving the “memory” of the autoimmune disease intact (96). In striking contrast, CD19 CAR-T cell therapy causes a complete disruption of follicular structures. FDCs acquire the CD19 protein directly from the surface membranes of interacting B cells through a process known as trogocytosis. By decorating their own surfaces with stolen CD19 antigens, the FDCs turn themselves into direct targets for the cytotoxic fury of the CAR-T cells (97). The eradication of the FDC network permanently erases the structural “hard drive” of the autoimmune disease. The fundamental histological and pharmacological differences between monoclonal antibody-mediated superficial depletion and CAR-T cell-mediated deep immune resets are summarized in **Table 3**.

**Table 3:** Mechanistic dichotomy: superficial depletion vs. deep immune reset in refractory autoimmunity.

Pharmacological/histological parameter	Monoclonal antibody (rituximab)	Cellular immunotherapy (CD19 CAR-T)	Ref.
Target Antigen	CD20 (Lost upon terminal differentiation)	CD19 (Expressed broader across lineage, capturing early plasmablasts)	(66, 107)
Mechanism of Cytotoxicity	Passive (Relies heavily on host complement CDC and effector cell ADCC/ADCP)	Active (Direct, serial perforin/granzyme-mediated cell lysis)	(11, 109)
Peripheral Blood Clearance	Profound (>95% clearance within weeks)	Absolute (100% clearance rapidly achieved)	(52, 114)
Solid Tissue Penetration (Synovium/Lymph Nodes)	Superficial (Fails to penetrate dense microenvironments; heavily inhibited by local BAFF)	Profound (Actively extravasates and infiltrates deep tissue sanctuaries)	(64, 114)
Follicular Dendritic Cell (FDC) Network	Entirely Preserved (Maintains autoimmune structural memory)	Completely Eradicated (Targeted via acquired CD19 trogocytosis)	(116, 117)
T Follicular Helper (TFH) Cells	Preserved	Eliminated (Due to the collapse of the B-cell/FDC signaling axis)	(114)
Long-Lived Plasma Cell (LLPC) Impact	Ineffective (LLPCs lack CD20; continuous autoantibody generation remains)	Spares mature CD19- LLPCs (Preserves pre-existing vaccine titers and protective immunity)	(71, 112)
Ultimate Clinical Outcome	Transient suppression; inevitable relapse upon memory B-cell repopulation	"Deep Immune Reset"; induces long-term, drug-free sustained remission	(8, 113)

The transition from passive pharmacological blockade to active cellular engineering fundamentally alters the depth of tissue depletion. The eradication of the FDC network by CAR-T cells represents the critical mechanistic threshold required to permanently delete immunological autoimmune memory. ADCC, Antibody-Dependent Cellular Cytotoxicity; ADCP, Antibody-Dependent Cellular Phagocytosis; BAFF, B-Cell Activating Factor; CAR-T, Chimeric Antigen Receptor T-cell; CDC, Complement-Dependent Cytotoxicity; FDC, Follicular Dendritic Cell; LLPC, Long-Lived Plasma Cell; TFH, T Follicular Helper Cell.

### 6.3 Tandem targeting and tolerogenic CAR-Tregs in RA

Translating these extreme cellular therapies to the highly localized synovial pathology of difficult-to-treat (D2T) RA introduces unique complexities. Early case reports highlight the necessity for aggressive, dual-targeting approaches. A 2024 study detailed the treatment of a highly active, seropositive D2T-RA patient utilizing an investigational, non-cryopreserved CAR-T cell therapy engineered with tandem receptors targeting both CD19 and CD20 antigens (98). Following infusion, the patient exhibited a dramatic collapse in rheumatoid factor levels, plummeting from 1,200 IU/mL to 13 IU/mL within one month, achieving a complete, drug-free clinical remission fully sustained through a 12-month follow-up (98).

For seronegative phenotypes or diseases where autoantibodies are not the exclusive pathogenic drivers, the field shifts toward tolerogenic strategies. Chimeric antigen receptor regulatory T cells (CAR-Tregs) are engineered to recognize specific autoantigens (such as citrullinated proteins in RA), migrate directly to the site of inflammation, and secrete highly potent immunosuppressive cytokines (IL-10, TGF-β) to enforce localized immune tolerance, completely sparing the systemic immune system from total ablation (99).

## 7 Conclusion

The immunological paradigm surrounding rheumatoid arthritis has irreversibly shifted. Rigorous primary transcriptomic and *in vivo* studies prove unequivocally that the presence of autoantibodies and autoreactive B cells does not equate exclusively to pathogenesis. Specific monoclonal anti-citrullinated protein antibodies possess dominant, targeted anti-inflammatory properties, while highly specialized subsets of regulatory B cells act as the ultimate arbiters of immune homeostasis, suppressing runaway T-cell proliferation. The widespread clinical failure of rituximab highlights the severe limitations of passive, unguided B-cell depletion. The survival of CD20-negative plasmablasts and the architectural fortification of tissue-resident memory B cells within the inflamed synovium dictate that peripheral clearance is an insufficient therapeutic endpoint.

Overcoming therapeutic resistance requires an absolute paradigm shift away from broad, untargeted systemic immune suppression and toward precision, tissue-guided spatial intervention. Stratifying patients via synovial RNA sequencing accurately identifies appropriate candidates for alternative pathway inhibition. For multidrug-refractory, B-cell-driven endotypes, the application of engineered living cellular therapies, notably CD19-directed CAR-T cells, represents the pinnacle of modern rheumatology. By physically penetrating deep tissue sanctuaries, actively eradicating long-lived plasma cell precursors, and forcing the total architectural collapse of pathogenic germinal centers, cellular immunotherapy executes a permanent, structural immune reset. This transition from chronic pharmacological palliation to definitive, curative-intent cellular engineering offers the first genuine pathway toward sustained, drug-free remission in severe autoimmune pathology.
